# The Orientation of the Prosthetic Trochlear Angle Is Predictable in Kinematically Aligned Total Knee Arthroplasty

**DOI:** 10.3390/jpm15020052

**Published:** 2025-01-28

**Authors:** Giorgio Cacciola, Daniele Vezza, Alessandro Massè, Luigi Sabatini

**Affiliations:** 1 Centro Traumatologico Ortopedico (C.T.O.), Department of Orthopaedics and Traumatology, University of Turin, 10124 Turin, Italy; alessandro.masse@unito.it; 2 Department of Robotic and Mini-Invasive Orthopaedic Surgery, Humanitas “Gradenigo” Hospital, 10153 Turin, Italy; daniele.vezza@unito.it (D.V.); luigi.sabatini@gradenigo.it (L.S.)

**Keywords:** kinematic alignment, MA_d_FC, prosthetic trochlear angle, lateral distal femoral angle, prediction

## Abstract

**Abstract: Objective**: This study aimed to predict the orientation of the prosthetic trochlear angle (PTA) relative to the quadriceps line of force (QLF) in kinematically aligned total knee arthroplasty (KA-TKA) by using preoperative radiographic parameters. **Methods**: This study included 144 patients who underwent KA-TKA with a femoral component designed for mechanical alignment (MA_D_FC), with a PTA of 6°. Radiographic parameters, including the lateral distal femoral angle (LDFA) and the QLF^FMA (quadriceps line of force–femoral mechanical axis angle), were measured pre- and postoperatively. We developed and validated a formula to predict PTA orientation based on these values: “X = QLF^FMA−(PTA−(90°−LDFA))”, where values of x > 0° predict a lateral PTA orientation, while x < 0° predicts a medial PTA. **Results**: The formula accurately predicted PTA orientation in 100% of the cases, with a difference between the predicted and actual PTA values of <0.5° in 75% of the cases. Patients with an LDFA < 86° and lower QLF^FMA values were identified as at risk for medial PTA orientation, which can affect patellar tracking. **Conclusions**: Our formula offers a reliable preoperative tool for predicting PTA orientation in KA-TKA, aiding in component selection and alignment strategies to improve patellofemoral function and patient outcomes.

## 1. Introduction

The evaluation of the patellofemoral joint is one of the most debated topics among surgeons performing total knee arthroplasty (TKA) through kinematic alignment (KA) [1,2]. Most femoral components (FCs) are designed to be implanted according to mechanical alignment (MA) philosophy. As a result, most femoral components designed for mechanical alignment (MA_D_FCs) have a prosthetic trochlear angle (PTA) of 6° oriented laterally to the mechanical axis [3,4]. This 6° angle is chosen to align the trochlear groove (TG) with the quadriceps force vector (QFV), ensuring that the QFV correctly acts on the patella. This alignment promotes proper patellofemoral tracking and supports the optimal function of the joint [4,5].

**Figure 1:**
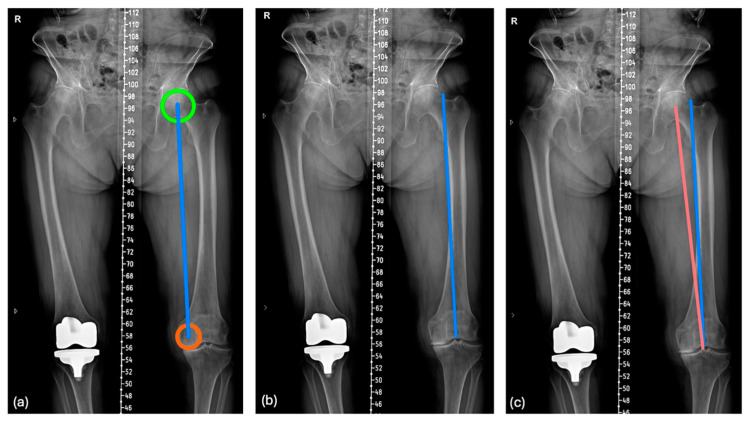
This figure illustrates the method used to determine the position of the QLF in a plain radiograph according to the method described by Tanifuji and Blaha [5]. The green circle represents the femoral head, the orange circle represents the medial side of the knee (**a**) Spherical axis (SA): the SA is a line that connects the best-fit circle of the femoral head to the center of the medial posterior femoral condyle. (**b**) QLF: the QLF is a line parallel to the SA that points to the center of the intercondylar notch. (**c**) QLF-FMA: the QLF-FMA is the angle between the QLF and FMA.

Several methods have been proposed to approximate the action of the QFV, given the complexity of representing a three-dimensional muscular vector on two-dimensional radiographs of the lower limbs [5,6,7,8]. Among these, the method described by Tanifuji et al. in 2012 has emerged as the most reliable representation. It has been validated in various subsequent studies [5,6] and is depicted on long-leg weight-bearing radiographs by the quadriceps line of force (QLF). The QLF, a line parallel to the spherical axis (SA), accurately represents the QFV (Figure 1).

If MA is chosen, the resulting alignment is highly reproducible on the coronal plane, as the goal of MA is to achieve a neutral postoperative alignment (all patients will have a lateral distal femoral angle (LDFA) and a medial proximal tibial angle (MPTA) of 90°) [5,9].

**Figure 2:**
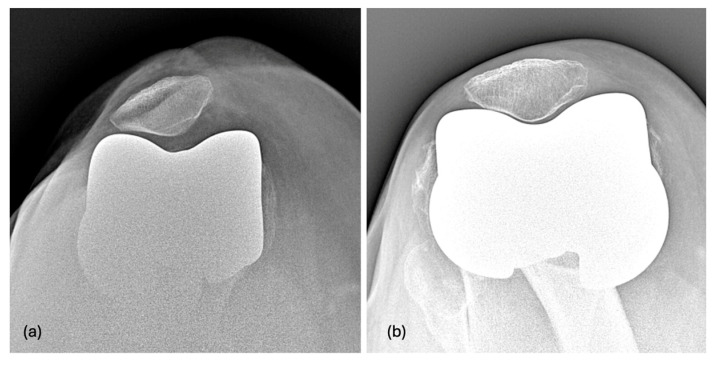
Difference between the patellar tilt of a mechanical alignment-designed femoral component (PTA = 6°) and a kinematic alignment-designed femoral component (KA_D_FC) (PTA = 20.6°): (**a**) Postoperative 30° axial radiograph of an unrestricted KA mechanical alignment-designed femoral component with a prosthetic trochlear angle of 6°. The prosthetic trochlear angle is medial to the QLF. As observed in the radiograph, the patella appears to be in a position of external patellar hyperpressure. (**b**) Postoperative 30° axial radiographs of an unrestricted KA-designed femoral component with a prosthetic trochlea angle of 20.6°. The prosthetic trochlear angle is lateral to the QLF. As observed in the radiograph, the patella is centered in the trochlear groove.

When KA is chosen, the femur undergoes pure distal and posterior resurfacing, indicating that the preoperative LDFA and the posterior condylar axis remain unchanged [10,11]. Since LDFA values vary across the population, using an MA_D_FC with a 6° PTA leads to less predictable outcomes in postoperative PTA orientation. In some cases, the postoperative PTA can be lateral to the QLF, ensuring proper quadriceps muscle action on patellofemoral joint function (Figure 2) [3]. In other cases, the PTA can be medial to the QLF, which can cause the quadriceps muscle to act at a disadvantage on the patella, potentially leading to lateral patellar hyperpressure, subluxation, dislocation, or patient discomfort (Figure 2) [12,13].

Recently, Howell’s group demonstrated that in a significant percentage of cases, the PTA is medial to the QLF when an MA_D_FC (with a PTA of 6°) is used to perform KA-TKA [3,4,5,6,7,8,9,10,11,12,13,14,15]. In a study involving 147 KA-TKAs, they found that in 86% of cases, the PTA was medial to the QLF. Moreover, these patients reported a postoperative forgotten joint score (FJS) that was 17 points lower than that in patients whose PTA was lateral to the QLF [14]. In a subsequent study, Jeremic et al. [15] observed that when a prosthesis designed for MA with a 6° PTA is used, the PTA is medial to the QLF in 51% of cases. They also found that the percentage of patients with a medial PTA relative to the QLF varies across different coronal plane alignment of the knee (CPAK) groups. Patients in CPAK groups III and VI exhibited a higher incidence of medial PTA relative to the QLF (89% and 50%, respectively) compared to other groups (CPAK I: 29%, CPAK II: 35%, CPAK IV: 0%, and CPAK V: 4%). Additionally, they reported that the CPAK III group had an average postoperative FJS of 43, which was significantly lower than the average FJS of other groups. In both studies, the position of the PTA relative to the QLF was measured retrospectively using postoperative long-leg radiographs of the knee.

These data demonstrate the importance of the LDFA in predicting the postoperative orientation of the PTA: patients with a CPAK III or VI phenotype, characterized by valgus alignment, have a higher risk of presenting with a PTA in an unfavorable position relative to the QLF compared to neutral and varus CPAK phenotypes (CPAK I, II, IV, and V). However, not all patients with a valgus phenotype will have the PTA medial to the QLF, just as not all other phenotypes will have a PTA lateral to the QLF. Therefore, being able to calculate the position of the PTA relative to the QLF prior to surgery can have significant implications for implant selection or alignment strategy [14,15]. Based on these important clinical findings, we developed a mathematical formula that predicts the position of the PTA in relation to the QLF. This prediction is based on a preoperative weight-bearing radiograph of the lower limbs and considers the LDFA, the PTA of the FC being used, and a new angle introduced for the first time in this study: the angle between the femoral mechanical axis (FMA) and the QLF named QLF^FMA. The primary aim of this study was to validate the accuracy of this formula to predict the orientation of the PTA relative to the QLF by comparing the predicted values with actual values measured on postoperative radiographs.

## 2. Materials and Methods

### 2.1. Study Design

After obtaining institutional review board approval (CET Interaziendale AOU Città della Salute e della Scienza di Torino 0055862), we retrospectively analyzed our internal arthroplasty registry (prospectively collected clinical and radiographic data) of patients who underwent primary KA-TKA with the use of MA_D_TKA with a 6° PTA (GMK Sphere, Medacta International) between April 2023 and April 2024. The same surgical technique (traditional caliper unrestricted KA) was used in all patients [11,16]. In all cases, the posterior cruciate ligament was excised, and in no cases was the patella resurfaced [17,18]. We included patients with diagnosis of primary knee osteoarthritis only and that had adequate preoperative and postoperative long-leg weight-bearing radiographs. To evaluate the validity of the mathematical formula, a power analysis established that at least 134 patients were required to obtain a power analysis of 0.80 with an alpha error of 0.05 and an effect size of 0.05.

### 2.2. Preoperative Radiographic Measurement

**Figure 3:**
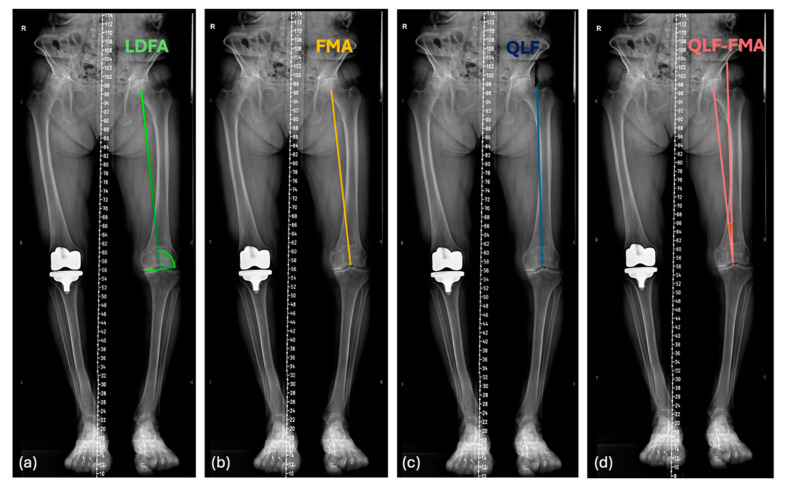
(**a**) Lateral distal femoral angle (LDFA); (**b**) femoral mechanical axis (FMA); (**c**) quadriceps line of force (QLF) measured according to Tanifuji et al.’s [5,6] method; (**d**) quadriceps line of force–femoral mechanical axis angle (QLF-FMA).

**Figure 4:**
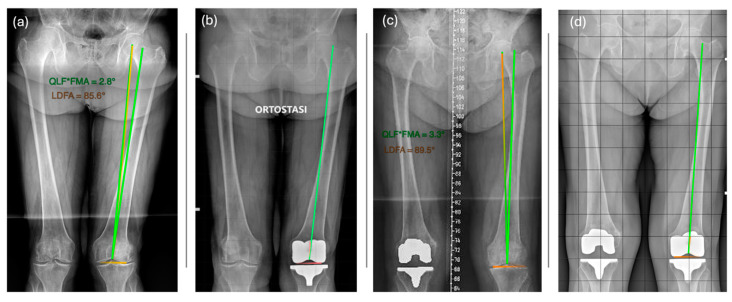
(**a**) In a patient with a QLF^FMA of 2.8° (green) and an LDFA of 85.6° (orange), we expected that the postoperative position of the trochlear groove of a femoral component with a PTA of 6° (orange) would be medial to the QLF (green) according to the mathematical formula of 1.2°. (**b**) Postoperative radiograph of the same patient, in which the angle measured on the long-leg radiograph of the lower limb was 1.2° medial to the QLF. (**c**) In a patient with a QLF^FMA of 3.3° and an LDFA of 88.5° we expected that the postoperative position of the trochlear groove of the femoral component with a PTA of 6° (orange) would be lateral to the QLF (green) according to the mathematical formula of 1.2°. (**d**) Postoperative radiograph of the same patient, in which the angle measured on the long-leg radiograph of the lower limb was 1.2° lateral to the QLF.

Two observers (G.C. and D.V.) measured all radiographic parameters on the preoperative and postoperative long-leg radiographs (Figure 3). The following angles were measured preoperatively: the lateral distal femoral angle (LDFA, that is, the lateral angle formed between the femoral mechanical axis and the joint line of the distal femur, Figure 3a), a new angle that was first described in this study; the quadriceps line of force–femoral mechanical axis angle (QLF^FMA, Figure 3d), that is, the angle between the line of force of the quadriceps muscle (QLF, Figure 3b) and a line that represents the femoral mechanical axis (FMA, going from the center of the femoral head to the center of the knee, Figure 4c) [5]; the angle formed between the distal joint line and the future prosthesis trochlear angle (PTA) (that is, 6° lateral of the femoral mechanical axis for the MAdFC used in this study) (Figure 1); and the theoretical angle formed by the expected PTA and the QLF after unrestricted KA-TKA.

### 2.3. Mathematical Formula for the Prediction of Postoperative PTA Orientation

We developed a mathematical formula that predicts the orientation (medial or lateral) and the value of the angle between the postoperative PTA and QLF. These equations seem to relate the distal femoral joint line to the forces exerted by the quadriceps, which is important for patellofemoral joint functionality.

X = QLF^FMA − (PTA − (90° − LDFA))(1)

The PTA is a value known by the manufacturer of the implant; in this study we used an FC designed for MA (GMK Sphere by Medacta International, Castel San Pietro, Switzerland) that has a PTA of 6. The “90-LDFA” is the difference between 90° and the LDFA, which indicates the deviation of the coronal femoral orientation from a mechanical cut. “PTA-(90-LDFA)” represents the deviation of the PTA from the FMA. By subtracting from the angle QLF^FMA, we obtain the PTA deviation relative to the QLF. Positive values indicate valgus alignment, whereas negative values indicate varus alignment. If X assumes a value < 0, the PTA will be medial to the QLF, whereas if X assumes a value > 0, the PTA will be lateral to the QLF [9,10]. If we know the PTA of the femoral component that we want to implant (assuming it is 6°, as in most prosthetic components designed for MA), we can calculate for each LDFA angle the values of the QLF-FMA for which the variable x will assume values greater than or less than zero. The formula application is simple, and measuring the parameters takes only a few minutes. A demonstration video is included as Supplementary Material.

### 2.4. Postoperative Radiographic Measure

Two different authors (G.C. and D.V.) measured the PTAs of MAdFC TKAs from 2-month-postoperative weight-bearing long-leg radiographs. A positive value indicates a PTA lateral to the QLF, whereas a negative value indicates a PTA medial to the QLF (Figure 4). Finally, we evaluated the difference between the predicted and measured values considering a value < 0.5° as optimal, a difference between 0.5° and 1° as acceptable, and a difference > 1° as poor.

### 2.5. Statistical Analysis

All statistical analyses were performed using Prism 7.0 for Mac OS, and power analysis was performed using G*power software. Continuous variables were expressed as means and standard deviations, while categorical variables were presented as frequencies and percentages.

To assess the relationship between the predicted values from the equation and the measured values from the radiographs, Pearson correlation analysis was performed. The predicted data were obtained by applying the specific formula (QLF^FMA − (6 − (90 − LDFA)) and were compared with the measured values collected directly from the postoperative radiographs. The Pearson correlation coefficient (*r*) was calculated to determine the strength and direction of the linear relationship between the two datasets. Statistical significance was set at *p* < 0.05.

The intraclass correlation coefficient (ICC) was calculated to evaluate both inter-observer and intra-observer reliability. For inter-observer reliability, measurements were independently performed by two observers (G.C. and D.V.) on the same set of radiographs. The ICC model was used to assess the consistency of ratings across different observers. For intra-observer reliability, a single observer repeated five randomly selected measurements on two separate occasions, and the ICC model was used to assess the stability of measurements over time by the same observer. ICC values were interpreted based on the following scale: poor reliability (<0.5), moderate reliability (0.5–0.75), good reliability (0.75–0.9), and excellent reliability (>0.9).

## 3. Results

### 3.1. Preoperative Clinical and Radiographic Data

After applying the inclusion and exclusion criteria, 144 patients (144 TKAs) were included in the study (GMK Sphere, Medacta International). The average age at the time of surgery was 75.1 years (range, 62–80 years), with 92 women (67.6%) and 44 men (32.4%), as well as 66 patients (48.5%), respectively. The diagnosis was confirmed in 100% of the cases. The mean preoperative HKA was 175.4° ± 3.3° (range, 164–180°). The average preoperative LDFA was 88.4° (range, 84–93°), and the average preoperative MPTA was 88.2° ± 2° (range, 85–93°). The average preoperative aHKA was 0.37°± 2.7 (range, −4°–5°). The average preoperative QLF^FMA angle was 3.2° ± 0.4 (range, 2.1°–4.5°). Postoperative values are summarized in Table 1. There were no significant differences between the preoperative and postoperative radiographic values for the parameters analyzed (except for HKA).

### 3.2. Prediction of QLF Position According to the Preoperative Radiographic Data

According to the mathematical formula proposed in the present study, we reported that the average value of the equation was −0.9° ± 2.1° (range, −4.8° to 2.8°). The results of the equation were greater than 0 in 54 cases (37.5%) and less than 0 in the remaining 90 cases (62.5%).

### 3.3. Verification of the Mathematical Formula

The prediction (medial or lateral position of the PTA to the QLF) was correct in 100% of the cases. The average value of the angle between the postoperative PTA and the QLF measured in the long-leg weight-bearing postoperative radiographs was 1.1° ± 2.2°. In 108 cases (75%), the difference between the predicted and measured values was less than 0.5°. In 24 cases (16.7%), the difference was between 0.5° and 1°, while the difference was greater than 1° in 12 cases (8.3%).

### 3.4. Interclass and Intraclass Correlation Coefficient

The inter-observer reliability for the preoperative and postoperative measurements of the QLF-FMA angle demonstrated a high level of consistency between the observers. The ICC was calculated to assess agreement, yielding a value of 0.989, indicating excellent reliability. Similarly, there was a high level of consistency between observers for the angle measured between the PTA and QLF (0.977). The intra-observer reliability for the measurements resulted in an excellent level of agreement for both QLF^FMA (0.997) and for the angle between the PTA and QLF (0.996).

## 4. Discussion

This study demonstrates that the orientation of PTA KA-TKA can be predicted using a mathematical model based on preoperative long-leg weight-bearing lower limb radiographic measurements. By incorporating the LDFA and the QLF^FMA, the proposed formula reliably predicted the postoperative alignment of the PTA relative to the QLF, with high accuracy across a large sample. These findings indicate that this predictive model can serve as a valuable tool in preoperative decision-making about implant and alignment choices. The predictability of PTA orientation is particularly relevant for KA-TKA, where femoral components undergo resurfacing rather than the standardized alignment adjustments typical of MA techniques. This variability in alignment can lead to inconsistent PTA orientations with an MA_D_FC and, according to recently published clinical studies, to a lower postoperative FJS when the PTA is medial to the QLF [3,14,15]. Additionally, as demonstrated by the intra- and inter-observer correlation coefficients, the measurements were highly reproducible, both when performed by the same author at different times and when performed by different authors.

This study has several important limitations that need to be assessed. The first major limitation of this study is that although the mathematical formula is fully valid for calculating the correct position of the PTA relative to the QLF, the clinical results supporting the hypothesis of this study are limited, with data coming from a small patient sample [3,14,15]. In addition, this study evaluates the position of the patella relative to the coronal plane at 0° of flexion only, without considering the kinematic dynamics of the patellofemoral joint at different degrees of flexion. The second major limitation is based on the same constraints related to the calculation of the QLF reported by Tanifuji himself, namely that the CT scans were performed in a supine position at 0° knee extension and do not account for cartilage thickness or changes in femoral geometry on the sagittal plane [5,6]. The third major limitation is that the action of the quadriceps force vector on the patella is only one of many factors influencing the functionality of the patellofemoral joint. Important factors, such as the inability to properly restore, with current femoral prosthetic designs, the trochlear prominence and patella thickness, the extent of the anterior medial and lateral condylar height, or the asymmetry of the medial and lateral femoral condyles (including the flatter lateral condyle in early and mid-flexion), are not taken into account [19,20,21]. Additional studies considering data from the sagittal and coronal planes are necessary to confirm these findings. Other parameters, such as preoperative trochlear morphology, postoperative patellar tilt, and the axial orientation of the prosthetic component may also affect outcomes [19,20]. Another important limitation is that the formula is valid only if the distal femoral cut is performed correctly. If the distal femoral cut is executed improperly, with asymmetry between the medial and lateral distal femoral condyles, the postoperative LDFA will vary, and consequently, the orientation of the PTA relative to the QLF will be affected. In this case, the actual position of the PTA relative to the QLF will need to be recalculated based on the new LDFA. As is typical with retrospective studies, there is also an inherent limitation due to the inability to control for all potential confounding variables. Moreover, the validity of the mathematical formula was only evaluated using a single femoral component design. In the future, the effectiveness of the formula will need to be tested using prostheses with different PTA values or alignment techniques. Lastly, the accuracy of predicting the positioning of the PTA relative to the QLF depends on the quality of the preoperative and postoperative radiographs [22,23]. Specifically, in cases where the abduction angle of the operated lower limb differs between the preoperative and postoperative radiographs, this can lead to discrepancies in the measured values (for this reason, 10 patients were excluded during recruitment due to poor image quality). This issue can be exacerbated in cases where a significant axis deviation is caused by severe joint wear [22,23].

Considering that the number of surgeons opting to implant knee prostheses using KA is steadily increasing, along with the total number of KA implants performed globally each year, knowing the position of the PTA relative to the QLF in advance can be a key factor in choosing the implant or alignment type [24,25]. Although kinematic alignment has proven to be an effective technique for resurfacing the distal femur and the posterior condylar axis, the same cannot be said for the anterior compartment [19,26,27]. Barroso et al. [19] analyzed three-dimensional reconstructions of a large series of osteoarthritic knees (4116 CTs from the 360 Knee System database), demonstrating that the native trochlear angle showed a wide variation from 23.8° of varus to 30.3° of valgus. In addition, they analyzed the “anatomy” of 45 different femoral components, demonstrating that the PTA will match the native trochlear angle only in 58.55° of cases if all the implants are available for implantation in the operating room. Similar results were also reported by Hazratwala et al. [26], who worked on the same dataset from the 360 Knee System Database. Modern femoral components are designed to facilitate the congruence of a patellofemoral joint showing a valgus trochlear groove (most of them have a PTA between 3° and 10° valgus) [19]. When MA is performed, the FC is positioned at 3° of external rotation relative to the posterior condylar axis to improve patellofemoral kinematics, avoiding maltracking and possible complications [26]. However, current MA_D_FCs fail to restore the trochlear geometry in KA, causing in most cases an excessive valgus orientation and proximal understuff with respect to the native articular surface [12,13], potentially leading to quadriceps muscle overuse/fatigue and increased joint reaction forces at the patellofemoral articular surface [12,13]. According to the results reported by Riviere et al. [13], when a 6° PTA MA_D_FC (Persona© by Zimmer) is kinematically aligned, attempting to restore the height of the lateral facet of the trochlea by increasing the thickness of the femoral component results in significant patellar overstuffing and mediolateral overhang [13]. One of the possible solutions is to implant a femoral component designed for kinematic alignment (KA_D_FC). In a recent study, Sappey-Mariner et al. [3] compared the position of the PTA relative to the QLF by comparing 35 bilateral TKAs, implanting an MA_D_FC (GMK Sphere, PTA = 6°) on one side and a KA_D_FC (GMK SpheriKA, PTA = 20.6°) in the contralateral knee. They observed that the PTA was lateral to the QLF in all cases in which the KA_D_FC was used but was lateral in 69% of knees only when an MA_D_FC with a 6° PTA was used. Despite the theoretical advantages of using a prosthetic design with a wider trochlear groove, they failed to report a statistically significant difference in clinical outcomes. Another advantage of using a KA_D_FC is the possibility to improve the lateral coverage of the anterior femoral resection compared to that achieved with an MA_D_FC [27].

In this study, we introduced a new angle, the QLF^FMA, which quantifies how lateral the quadriceps force vector is relative to the FMA. Larger QLF^FMA values indicate a more lateral vector, while smaller values indicate a less lateral vector. The average QLF^FMA observed in this study, based on a sample of 144 knees, was 3.2 ± 0.4°, with values ranging from 2.1° to 4.5°. This implies that for a given LDFA, patients may exhibit a PTA that is either medial or lateral relative to the QLF. To clarify, consider two examples:

- Patient A has an LDFA of 87° and a QLF^FMA of 2°.
- Patient B also has an LDFA of 87° but a QLF^FMA of 4°.

Both patients received the same femoral component with a PTA of 6°. Substituting these values into the equation yields the following:

- For Patient A, the QLF is lateral to the PTA by 1° (x = 2 − (6 − (90 − 87))).
- For Patient B, the PTA is medial to the QLF by 1° (x = 4 − (6 − (90 − 87))).

Table 2 summarizes the QLF^FMA values for which the PTA will be medial or lateral relative to the QLF for a given LDFA, assuming the use of a prosthesis with a trochlear angle of 6°. Based on the QLF^FMA values observed in this study (minimum 2.1°), we can conclude the following: if a prosthesis with a 6° trochlear angle is implanted, the PTA will be medial to the QLF in any case where the LDFA is 85° or less. Conversely, in patients with an LDFA of 90° or greater, the PTA will always be lateral to the QLF (as the maximum observed QLF^FMA value was 4.5°). For patients with an LDFA between 85° and 90°, preoperative knowledge of the QLF^FMA value could be crucial in predicting the postoperative orientation of the PTA if unrestricted KA is chosen. In cases where the PTA is expected to be medial to the QLF, alternative alignment techniques or prostheses with a greater PTA can be considered. Our findings align with a recent report by Jeremic et al. [15], who noted that CPAK groups III and IV (valgus morphotypes) are at a higher risk of the PTA being medial to the QLF, with this occurring in 89% and 50% of cases, respectively, compared to varus and neutral morphotypes. The use of femoral components with a greater PTA, such as the GMK SpheriKA (PTA = 20.6°), could potentially eliminate the risk of the PTA being medial to the QLF. Despite the theoretical advantage of using a KADFC to enhance patellofemoral kinematics and improve clinical outcomes, current evidence remains limited [3,14,15].

## 5. Conclusions

Our findings provide significant insights into the preoperative prediction of the position of the PTA relative to the QLF by determining the LDFA and QLF^FMA. Consistent with the work of Howell et al. [9] and Sappey-Marinier et al. [3], our study highlights the critical role of accurately predicting PTA orientation in KA-TKA.

Howell et al. demonstrated that a lateral PTA orientation relative to the QLF is associated with significantly improved patient-reported outcomes, including a 17-point increase in FJS compared to that for medial orientations. Similarly, Sappey-Marinier et al. emphasized that the KADFC reliably achieved lateral PTA alignment.

Our predictive model based on the QLF^FMA (ranging from 2.1° to 4.5°) confirms that for patients with a valgus femur angle of 3° or greater, selecting a femoral component with a wider trochlear groove ensures lateral PTA positioning relative to the QLF. This model serves as a practical preoperative tool for selecting femoral components and alignment strategies to optimize patellar tracking and improve functional outcomes in KA-TKA [3,9].

Although this study employs a formula to predict PTA orientation in patients undergoing KA-TKA, it can also be applied in different alignment strategies. By substituting the LDFA value with the “predicted” value for alternative alignments, such as MA (where the LDFA is set at 90°), or the planned value in cases of restricted KA, the formula remains applicable across these varied contexts.

Further research involving larger patient cohorts and adequate follow-up is necessary to validate these findings and their clinical implications.

## Figures and Tables

**Table 1:** Preoperative and postoperative radiographic parameters. The only significant difference was noted for HKA, whereas no differences were observed for the other values.

Variable	Preoperative	Postoperative	*p*-Value
HKA	175.4 ± 3.3°	177.4° ± 2.4°	<0.001
LDFA	88.2° ± 2°	87.9° ± 1.8°	0.1820
MPTA	87.3° ± 2.1°	87.1° ± 2°	0.4086
aHKA	0.37° ± 2.7°	0.55° ± 3.1°	0.5796
QLF^FMA	3.2° ± 0.4°	3.1° ± 0.4°	0.9854

**Table 2:** Prediction of the position of the PTA compared to the QLF according to the LDFA value based on the QLF^FMA value for a prosthesis that has a PTA of 6°.

	For Prosthesis with a 6° PTA	
LDFA	PTA Medial if QLF^FMA:	PTA Lateral if QLF^FMA:
84°	<0°	>0°
85°	<1°	>1°
86°	<2°	>2°
87°	<3°	>3°
88°	<4°	>4°
89°	<5°	>5°
90°	<6°	>6°
91°	<7°	>7°
92°	<8°	>8°
93°	<9°	>9°
94°	<10°	>10°
95°	<11°	>11°
96°	<12°	>12°

## Data Availability

The original contributions presented in this study are included in the article. Further inquiries can be directed to the corresponding author.

## Supplementary Materials

The following supporting information can be downloaded at: https://we.tl/t-iWSHLk43go (accessed on 18 January 2025).
